# Association of eHealth Literacy with Health Promotion Behaviors of Community-Dwelling Older People: The Chain Mediating Role of Self-Efficacy and Self-Care Ability

**DOI:** 10.3390/ijerph19106092

**Published:** 2022-05-17

**Authors:** Yinuo Wang, Yuting Song, Yaru Zhu, Heqian Ji, Aimin Wang

**Affiliations:** School of Nursing, Qingdao University, Qingdao 266100, China; wangyinuo1208@163.com (Y.W.); yuting.song@ualberta.ca (Y.S.); 17853462637@163.com (Y.Z.); jhq15935115077@163.com (H.J.)

**Keywords:** older people, eHealth literacy, self-efficacy, self-care ability, health promotion behaviors

## Abstract

In the digital age, electronic health literacy (eHealth literacy) of community-dwelling older people plays a potentially important role in their health behaviors which are critical for health outcomes. Researchers have documented that self-efficacy and self-care ability are related to this relationship. This study aimed to assess the relationship between eHealth literacy and health promotion behaviors among older people living in communities and explore the chain mediating role of self-efficacy and self-care ability. For this cross-sectional study, we used data from 425 older adults at 3 communities in Qingdao, Shandong Province in Northeastern China, from June to September 2021. Path analysis using the structural equation model was performed. We found that eHealth literacy was significantly associated with health promotion behaviors in older people. Additionally, eHealth literacy indirectly affected health promotion behaviors through self-efficacy and self-care ability, respectively. In addition, the chain mediation effect was identified in the relationship of eHealth literacy and health promotion behaviors: eHealth literacy→ self-efficacy→ self-care ability→ health promotion behaviors. These findings offer promising directions for developing interventions to modify older adults’ health behaviors through enhancing their eHealth literacy. These interventions should integrate components that target improving the self-efficacy and self-care ability of older people.

## 1. Introduction

Population aging is a global issue. In China, in particular, the population of people aged over 65 has reached 176.03 million, accounting for 12.6% of the total population in China [1]. The health status of older people is of great importance to the healthy development of society. However, the surge in the older adult population poses a challenge to the health care system, as older people face unique challenges with health problems, such as higher rates of physical and mental health issues [2,3]. However, in the face of various health problems, few older people adopt effective health behaviors [4]. Research shows that the level of health promotion behaviors of older people needs to be improved [5]. Lacking health promotion behaviors can threaten the health of older people [6]. Therefore, encouraging older people to adopt healthy behaviors is conducive to improving the health status of older adults and reducing the health cost for society. 

Many factors influence the (non)adoption of health promotion behaviors, such as self-efficacy, social support, health concept, and health knowledge [7,8]. Health literacy, the ability of individuals to actively access information that is beneficial to them, has a significant influence on health promotion behaviors [9]. Research shows that high health literacy can facilitate individuals’ understanding of disease severity and adoption of disease prevention behaviors [10,11]. Recently, during the COVID-19 pandemic, health literacy has influenced both vaccination hesitation and adherence to preventive measures of the general public [12,13]. Therefore, many scholars have recommended helping individuals adopt health promotion behaviors by improving their health literacy [14,15].

In recent years, the convenience and low cost of the internet have enabled it to be an important way to export health knowledge, therefore, researchers began to pay attention to electronic health literacy (eHealth literacy), defined as the ability to search, find, understand, and evaluate health information through network resources and use the acquired information to process and solve health problems [16]. The effect of eHealth literacy on health has attracted significant attention from researchers [17]. Studies have shown that patients with higher eHealth literacy are more likely to manage their health by using the information obtained from the network [18], and poor eHealth literacy can hinder patients’ ability to manage their medications [19]. In the process of medical decision-making, patients strive to improve their electronic health literacy to manage their health [20]. Especially during the time of COVID-19, the relationship between eHealth literacy and health behaviors has been widely explored [21,22]. People with extensive experience in using the internet are more protective and aware of the importance of taking proactive health measures after an illness has occurred.

Studies have demonstrated the positive effects of eHealth literacy on health among the general population. However, older adults face unique challenges when using the internet, such as poor eyesight and limited mobility, which may affect their interest in using the internet and reduce the frequency of internet use [23]. Moreover, most older adults use the internet to socialize and get news, instead of obtaining health information. Approximately 40% of elderly participants have used the internet to access health information [24,25]. Therefore, the relationship between eHealth literacy and health promotion behaviors in the older population needs to be further explored. 

Previous studies have also indicated that eHealth literacy can influence health promotion behaviors through self-care ability and self-efficacy, respectively [20,26,27,28], but the interactions among these pathways have not been examined. In this study, we aimed to address the gaps by focusing on the mechanisms by which eHealth literacy influences health promotion behaviors. Particularly, we focused on the correlation of eHealth literacy, self-efficacy, self-care ability, and health promotion behaviors, and the relationship among them. 

## 2. Research Model and Hypotheses 

We drew on the Knowledge, Attitude, and Behavior (KAB) model and the Health empowerment theory.

Firstly, the KAB model is a theoretical model of behavioral intervention aimed at changing human health behaviors [29]. According to the model, knowledge refers to the process of individuals receiving health knowledge and is the basis of their health behavior change. Beliefs are attitudes based on the individual’s reflection on the knowledge received, which is gradually transformed into positive beliefs and is the driving force behind the individual’s health behavior change. Behavior is action, which refers to the transformation of health-damaging behaviors by individuals who uphold positive beliefs and attitudes based on their acquired and perceived health knowledge [30]. The KAB model proposes that knowledge can directly and indirectly influence behavior by changing one’s beliefs [31]. In previous studies, self-efficacy has often been used to measure individuals’ beliefs. The information people gain from the internet will increase their knowledge and improve their self-efficacy [32]. At the same time, some studies have shown that the self-efficacy of older people may be related to eHealth literacy. Older people with lower eHealth literacy are less able to access, use, and judge online health information, which may affect their confidence and attitudes to change health behaviors [33].

Secondly, in recent years, Fotoukian and colleagues [34] developed the Health Empowerment theory as the process by which patients actively develop and utilize their knowledge and abilities, develop confidence, gain self-development and self-satisfaction, and increase their sense of self-efficacy to manage their illness, manage their lives, and promote their health. Health empowerment theory suggests that health behavior change requires improving an individual’s capacity for self-care [35]. The key to improving self-care ability is to encourage individuals to make full use of their knowledge and other available resources [36]. The information that older people access from the internet is an important health resource [37]. Moreover, an individual’s sense of healthy beliefs is an important psychological resource.

**Figure 1:**
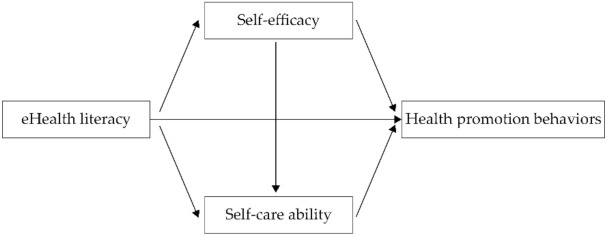
The conceptual model.

Drawing on the KAB model and the Health Empowerment theory, we developed a conceptual model for our study (Figure 1). 

**Our hypotheses were:**

**Hypothesis** **1** **(H1).** *Older people’s health promotion behaviors increase with the increase of their eHealth literacy.*

**Hypothesis** **2** **(H2).** *Older people’s eHealth literacy indirectly influences health promotion behaviors through self-efficacy.*

**Hypothesis** **3** **(H3).** *Older people’s eHealth literacy indirectly influences health promotion behaviors through self-care ability.*

**Hypothesis** **4** **(H4).** *Older people’s eHealth literacy influences self-care ability by affecting self-efficacy, and ultimately affects health promotion behaviors.*

## 3. Materials and Methods

### 3.1. Study Design, Settings, and Participants

The data used in this study were obtained from a multicenter cross-sectional survey that investigated patients’ eHealth literacy in Qingdao, Shandong Province, China, from June to September 2021. Patients were selected from a convenience sample of three communities. Older adults were eligible if they were aged ≥ 60 years and were able to communicate. We excluded persons with severe mental illness, severe hearing or visual impairment, or severe physical illness that prevented them from participating in this study. Additionally, we excluded people who dropped out during the study or answered questionnaires incompletely. 

### 3.2. Data Collection Procedure 

One of the authors (A.W.) established a long-term research collaboration with the three communities. She first contacted the community health service center managers to explain the purpose and significance of the study and to obtain their consent and cooperation. Then, two uniformly trained researchers (Y.W. and Y.Z.) recruited participants and collected data onsite. Potential participants were older people who visited the community health service center during the data collection period. After explaining the purpose and significance of the study and obtaining consent, the questionnaires were distributed to participants and the participants were informed of the precautions and requirements for completing the questionnaires in a standardized instructional language. For those who needed help, the researchers read out the questionnaires word by word and then filled out the questionnaires for them according to their wishes and understanding. Most participants completed the questionnaire at the end of their visit and a few completed it before their visit. To thank participants for their participation in the study, the researchers offered small gifts to each participant (e.g., hand cream, shopping bags). Confidentiality was strictly observed throughout the process. The questionnaires were distributed, collected, and checked on the spot, and any missing items were completed in time. We approached 500 older adults and 450 agreed to participate. In total, 425 older people completed the questionnaire.

### 3.3. Ethical Consideration

This study was approved by the institutional review board of Qingdao University (reference ID: 2021115). One of the researchers approached potential participants to explain the study. Potential participants who agreed to participate in the study took approximately 15–20 min to fill out the survey.

### 3.4. Measures

#### 3.4.1. eHealth Literacy Scale (eHEALS)

We used the eHEALS to measure older peoples’ knowledge, comfort, and perceived skills of searching for, evaluating, and applying eHealth information to manage health problems [38]. The eHEALS has 8 items rated on a 5-point Likert scale (1 = strongly disagree, 5 = strongly agree) and 3 dimensions (application ability (AB), decision-making ability (DMB), judgment ability (JA)). The sum score ranges from 8 to 40, with a higher score indicating greater perceived eHealth literacy. Researchers have used this scale among older patients with cancer and older adults visiting medical clinics, reporting high reliability and validity among the older adult population [27,39]. We used a modified Chinese version of eHEALS in this study [40]. The Cronbach’s α coefficient of eHEALS was 0.884 in our sample.

#### 3.4.2. General Self-Efficacy (GSE)

To measure older adults’ self-efficacy, we used the GSE scale [41]. The scale has 10 items, and composite scores for GSE range from 10 (low GSE) to 40 (high GSE). The scale has been used in numerous research studies, where it yielded good to excellent internal consistency with Cronbach’s α from 0.75 to 0.91 [42]. In this study, the Cronbach’s α coefficient of GSE was 0.789.

#### 3.4.3. Health-Promoting Lifestyle Profile-II, Revised (HPLP-II R)

We used the Health-Promoting Lifestyle Profile (HPLP) to measure health promotion behaviors [43]. The revised version, HPLP-II, has been widely used with patients and healthy adults in China [44]. The revised instrument has 40 items with 6 subscales (health responsibility, pressure management, nutrition, physical exercise, interpersonal relationship, and spiritual growth). The total score ranged between 40 and 160; the higher the score, the better the health-promoting lifestyle. In this study, the Cronbach’s α coefficient was 0.817.

#### 3.4.4. The Self-Care Ability Scale for the Elderly (SASE)

In this study, we used the Chinese version of the SASE scale to measure older people’s self-care ability [45]. The scale consists of 17 items. A 5-point Likert scale was used, ranging from “completely disagree” to “completely agree”, on a scale of 1 to 5, with a total score range of 17 to 85. In this study, the Cronbach’s α coefficient of SASE was 0.767.

### 3.5. Data Analysis

We analyzed data using IBM SPSS 24.0 and AMOS 24.0. We calculated frequency and percentage for categorical variables, and mean and standard deviation for continuous variables. We used Pearson correlation analysis to assess the relationship between variables. Structural equation modeling was then conducted using AMOS 24.0 to test the mediating effect. We used χ^2^, χ^2^/df, goodness-of-fit index (GFI), comparative fit index (CFI), normed fit index (NFI), and root mean square error of approximation (RMSEA) to evaluate the goodness-of-fit of the model. To verify the statistical significance of the final structural equation model, we used critical ratio (CR) and *p* values [46]. The bootstrap maximum likelihood was used 2000 times within the 95% confidence interval to test the significance of the direct, indirect, and total effects of the model. Finally, the PROCESS program was further applied to conduct a Bootstrap method mediating effect significance test to verify whether the above model paths were valid. The level of statistical significance was set at 0.001.

## 4. Results

### 4.1. Participants’ Demographics and Correlations among Variables

Table 1 shows the demographic characteristics of participants in our sample.

Table 2 presents the mean value, standard deviation, and correlation coefficient of the variables of interest in this study. The results show a significant positive relationship between eHealth literacy and health promotion behaviors and its various dimensions (*r* = 0.188~0.519, all *p <* 0.01), supporting H1. Additionally, there is a significant positive correlation between eHealth literacy and self-efficacy, self-care ability, respectively (*r* = 0.476, *p <* 0.01; *r =* 0.497, *p* < 0.01).

### 4.2. The Structural Equation Model 

**Figure 2:**
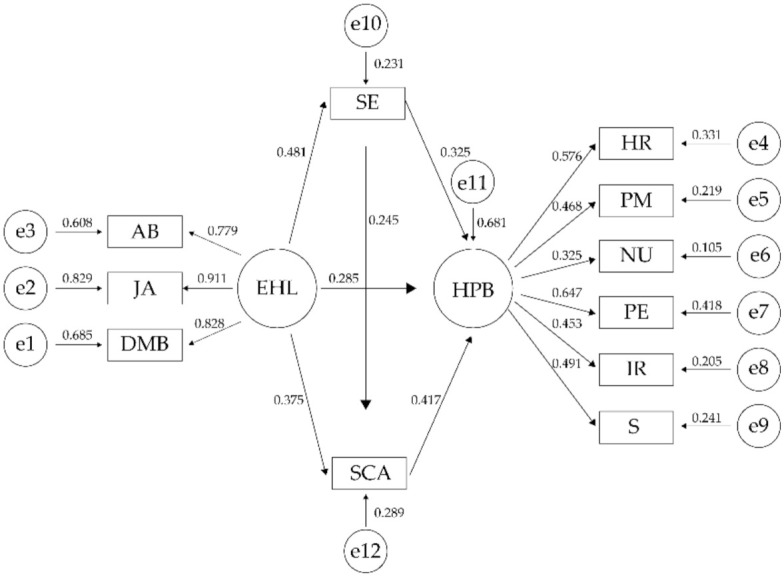
Path analysis model: eHealth literacy, self-efficacy, self-care ability, and health promotion behaviors. **Note.** EHL indicates eHealth literacy; AB indicates application ability; DMB indicates decision-making ability; JA indicates judgment ability; SCA indicates self-care ability; SE indicates self-efficacy; HPB indicates health promotion behaviors; HR indicates health responsibility; PM indicates pressure management; NU indicates nutrition; PE indicates physical exercise; IR indicates interpersonal relationship; S indicates spiritual growth. e1–e12 denote the measurement error of observed variables in estimating the latent variables.

We used IBM AMOS 24.0 to test our conceptual model of the relationships among eHealth literacy, self-efficacy, self-care ability, and health promotion behaviors. As Table 3 shows, the fit indices of the model all reach the standard level, which indicates that the model works well. Figure 2 shows that all direct paths were significant. eHealth literacy was a positive predictor of health promotion behaviors (*β* = 0.285, *p* < 0.001), self-efficacy (*β* = 0.481, *p* < 0.001), and self-care ability (*β* = 0.375, *p* < 0.001). eHealth literacy also indirectly influenced health promotion behaviors through self-efficacy (*β* = 0.156, *p* < 0.001) and self-care ability (*β* = 0.156, *p* < 0.001) receptively.

### 4.3. Significance Tests for Intermediation Effects

The PROCESS macro was used to examine the multiple mediating roles of self-efficacy and self-care ability in the relationship between eHealth literacy and health promotion behaviors. Bootstrap test for mediating effect was applied and 95% CI for mediating effect was calculated with a sample size of 2000 selected. The results are shown in Table 4. None of the 95% confidence intervals for the path coefficients included zero, suggesting that the total effects, direct effects, and indirect effects were all significant (0.519, 0.226, and 0.293, respectively). The mediating effect of self-efficacy was 0.132, accounting for 25.39% of the total effect, supporting H2; the mediating effect of self-care ability was 0.123, accounting for 23.77% of the total effect, supporting H3; the effect of the path ‘eHealth literacy → self-efficacy → self-care ability → health promotion behaviors’ was 0.038, accounting for 7.25% of the total effects, supporting H4.

## 5. Discussion

This study supported our hypotheses about the relationship among eHealth literacy, self-efficacy, self-care ability, and health promotion behaviors. Structural equation modeling suggested a direct effect of eHealth literacy on health promotion behaviors and an indirect effect on health promotion behaviors through self-efficacy and self-care ability. Our findings provide potential directions for interventions to facilitate health promotion behaviors in older people.

The study showed that the average score of eHealth literacy among older adults in the community was (16.541 ± 4.177), which was lower than that of university students [47]. This aligns with findings from other studies of the older adult population [27], supporting the urgent need to improve the eHealth literacy of older people. Moreover, the average score of health promotion behaviors of older people in the community was at an intermediate level (101.659 ± 11.804), with stress management scores (12.261 ± 2.284) and spiritual growth scores (12.887 ± 2.345) being lower, indicating that there is room for further improvement in health promotion behaviors of older people, especially in the spiritual dimension, which could be further enhanced. 

### 5.1. The Direct Effect of eHealth Literacy on Health Promotion Behaviors

We found that eHealth literacy can directly contribute to health promotion behaviors. Our findings aligned with previous studies of people living with diabetes, and students, which reported that eHealth literacy helped people gain knowledge about glycemic index management and increased health awareness [48,49]. Based on the KAB theory, behavior is influenced by knowledge [31]. Strong internet literacy helps to broaden older people’s access to a wide range of health knowledge. Our findings suggested that community health workers and relevant personnel can promote the elderly to take active health behaviors by improving their eHealth literacy. Although limited, a few interventions are emerging that target eHealth literacy. Customized guidance based on individual characteristics is essential in the process of improving individual eHealth literacy. A study of rural cancer survivors preferred personalized help to standardized computer sessions [50]. It is also possible to make full use of media technology. A professionally developed tool consisting of web pages, videos, interactive games, and an online video course on how to access health websites both contributed to the eHealth literacy of users [51,52]. 

### 5.2. Indirect Effects of eHealth Literacy on Health Promotion Behaviors

KAB theory suggests that the production of behavior is not only influenced by knowledge, in which beliefs also play a key role, which is a crucial step in putting knowledge into action. In this study, self-efficacy not only directly influenced health promotion behaviors but also mediated the effect of eHealth literacy on health promotion behavior. One explanation is that self-efficacy refers to an individual’s evaluation and judgment of his or her abilities, and knowledge may be an important resource for an individual to measure his or her value. When an individual has more knowledge, the more confident the individual may be in changing undesirable behaviors. Huang, C.L. et al. showed that students with higher eHealth literacy had more positive views and beliefs about medication management [53]. Kyoung A Kim et al. found that eHealth literacy was an important influence on self-efficacy when they investigated how type 2 diabetes patients understood and used online health information related to health promotion behavior [54]. With higher levels of eHealth literacy, an individual’s ability to access information from the internet increased, enabling older adults to acquire more health knowledge, which can increase their self-efficacy [55]. These results further suggest that improving older adults’ self-efficacy is beneficial to their positive health outcomes.

Self-care ability also plays a mediating role in the effect of eHealth literacy on health promotion behaviors. Orem considered self-care ability as the competence to use various resources for self-care activities or self-management [56]. Our results showed a correlation between self-care ability and health promotion behaviors, which aligned with previous research findings. Older adults with high self-care ability may have higher levels of mental and life independence, resulting in a higher level of quality of life. Additionally, eHealth literacy was shown to be a significant predictor of self-care ability [37]. Results from a study in the US showed that approximately 75% of adults with chronic conditions had searched for health information on the internet. The high rates of information-seeking and use of internet-based health technologies reported by participants with chronic conditions may reflect the fact that eHealth contributed to self-management of chronic conditions [57]. This suggested that it was feasible to enrich an individual’s knowledge base through eHealth literacy interventions, ultimately improving self-care ability. However, the results of this study showed the score of self-care ability among older people in the community was only (60.341 + 6.989), which suggested that community health workers should make full use of digital technology to increase the knowledge base of older people related to the internet through online channels such as small programs, online consultations with community doctors, and health websites, and encourage older people to manage their health through the internet, which is conducive to improving older people’s health status.

### 5.3. The Chain Mediating Effects

The results of this study suggested that eHealth literacy improved older people’s self-efficacy, which then improved their self-care ability and ultimately, promoted good health behaviors. A study showed that patients with a higher sense of self-efficacy and greater resilience in overcoming difficulties showed a better implementation of self-care behaviors [58]. In this study, older people with higher self-efficacy were more likely to use the internet to look up health information and thus had more health knowledge, and used the knowledge gained to improve self-care when faced with health problems, ultimately, influencing health behaviors. This better explains the mechanism by which eHealth literacy influences health promotion behaviors and suggests that efforts are needed to promote older adults’ self-efficacy so they are actively involved in health management, while also improving their eHealth literacy.

### 5.4. Limitations and Recommendations

To the best of our knowledge, this study was the first to use structural equation modeling to explore the mechanisms underlying the effects of eHealth literacy on health promotion behaviors in older people, and the first to explore the relationship between self-care ability about eHealth literacy and health promotion behaviors. The findings of the study provide potential directions for interventions to facilitate health promotion behaviors of older adults. Certain limitations should be taken into consideration when interpreting the findings. Firstly, we used a cross-sectional design and thus, cannot conclude causal relationships between variables. Further research adopting longitudinal designs are warranted. In addition, we used convenience sampling and selected our sample from three communities in a Northeastern city in China, therefore generalization of findings to older adults with characteristics different from our sample require caution.

Our results have practical implications for community work and older people themselves. The model suggests that interventions for older people in the digital age should be multifaceted to reduce potential barriers to health promotion behaviors among older people. Self-efficacy plays an important role in the relationship between eHealth literacy and health promotion behaviors. Additionally, self-care ability is an important influencing factor of health promotion behaviors, thus, older people should be given a sense of full participation and autonomy throughout the intervention. This is conducive to increasing the initiative of achieving self-care and maintaining the long-term stability of good health behavior among the elderly.

## 6. Conclusions

We identified the association between eHealth literacy and health promotion behaviors among older adults and the mediating roles of self-efficacy and self-care ability. Our findings provide promising directions for interventions to promote health behavior interventions in the older adult population. Interventions should focus on improving eHealth literacy, and enhancing self-efficacy and self-care ability to achieve optimal health outcomes.

## Figures and Tables

**Table 1:** Participants’ demographics (*n* = 425).

Variable	Categories	*n*	%
Age	60–70 years	205	48.2
	71–80 years	172	40.5
	81–90 years	48	11.3
Marital Status	With spouse	357	84.0
	No spouse	68	16.0
Education level	Primary or no qualifications	68	16.0
	Secondary	201	47.3
	Graduates and above	156	36.7
Monthly income (RMB)	<1000	20	4.7
	1000–2000	39	9.2
	2000–3000	56	13.2
	3000–4000	116	27.3
	4000–5000	129	30.4
	>5000	65	15.3
Sex	Female	213	50.1
	Male	212	49.9

**Table 2:** Means, standard deviations, and correlations among variables.

	Mean	SD	EHL	AB	DMB	JA	SCA	SE	HPB	HR	PM	NU	PE	IR	S
EHL	16.541	4.177	1												
AB	10.988	2.652	0.949 **	1											
DMB	1.993	0.894	0.796 **	0.604 **	1										
JA	3.560	1.082	0.876 **	0.713 **	0.767 **	1									
SCA	60.341	6.989	0.497 **	0.478 **	0.394 **	0.420 **	1								
SE	24.734	4.129	0.476 **	0.442 **	0.437 **	0.393 **	0.425 **	1							
HPB	101.659	11.804	0.519 **	0.484 **	0.440 **	0.451 **	0.554 **	0.522 **	1						
HR	26.205	4.495	0.461 **	0.452 **	0.345 **	0.384 **	0.359 **	0.395 **	0.710 **	1					
PM	12.261	2.284	0.294 **	0.264 **	0.285 **	0.255 **	0.291 **	0.302 **	0.562 **	0.377 **	1				
NU	20.035	3.282	0.188 **	0.161 **	0.176 **	0.183 **	0.182 **	0.220 **	0.528 **	0.154 **	0.188 **	1			
PE	16.624	3.775	0.415 **	0.379 **	0.363 **	0.372 **	0.506 **	0.406 **	0.682 **	0.318 **	0.273 **	0.220 **	1		
IR	13.647	2.831	0.204 **	0.199 **	0.174 **	0.155 **	0.344 **	0.243 **	0.595 **	0.256 **	0.200 **	0.216 **	0.216 **	1	
S	12.887	2.345	0.264 **	0.237 **	0.234 **	0.244 **	0.331 **	0.321 **	0.569 **	0.252 **	0.190 **	0.167 **	0.167 **	0.370 **	1

**Note.** ** *p* < 0.01, *n* = 425; SD indicates “standard deviation”; EHL indicates eHealth literacy, measured by eHEALS scale scores. AB, DMB, and JA refer to the abbreviations of three dimensions of the eHealth Literacy Scale (Section 3.4.1). (AB indicates application ability; DMB indicates decision-making ability; JA indicates judgment ability; SCA indicates self-care ability); SE indicates self-efficacy, measured by GSE scale scores; HPB indicates health promotion behaviors, measured by HPLP-II R scale scores. HR, PM, NU, PE, IR, and S denote the scores of six dimensions of the HPLP-II R scale. (HR indicates health responsibility; PM indicates pressure management; NU indicates nutrition; PE indicates physical exercise; IR indicates interpersonal relationship; S indicates spiritual growth).

**Table 3:** Model fitting index.

Index	χ^2^/df	GFI	RMSEA	NFI	CFI
Result	2.846	0.955	0.066	0.927	0.951
Ideal value	<5.000	>0.900	<0.080	>0.900	>0.900

**Table 4:** Bootstrap analysis of multiple mediation effects.

	Effect Size	SE	95% CIs of Indirect Effect		Percentage of Total Effects (%)
			Lower Bound	Upper Bound	
Indirect effects	0.293	0.035	0.230	0.369	56.41
EHL **→** SE’ **→** HPB	0.132	0.025	0.083	0.183	25.39
EHL **→** SCA **→** HPB	0.123	0.024	0.079	0.174	23.77
EHL **→** SE’ **→** SCA **→** HPB	0.038	0.010	0.018	0.058	7.25

**Note.** EHL indicates eHealth literacy; SCA indicates self-care ability; SE’ indicates self-efficacy; HPB indicates health promotion behaviors; IR indicates interpersonal relationship.

## Data Availability

The datasets analyzed during the current study are not yet publicly available but are available from the corresponding author upon reasonable request.
